# Effect of Clotting Duration and Temperature on BDNF Measurement in Human Serum

**DOI:** 10.3390/ijms18091987

**Published:** 2017-09-15

**Authors:** Patrizia Amadio, Leonardo Sandrini, Alessandro Ieraci, Elena Tremoli, Silvia Stella Barbieri

**Affiliations:** 1 Centro Cardiologico Monzino, IRCCS, 20138 Milan, Italy; patrizia.amadio@ccfm.it (P.A.); leonardo.sandrini@unimi.it (L.S.); elena.tremoli@ccfm.it (E.T.); 2 Department of Pharmacological and Biomolecular Sciences, University of Milan, 20133 Milan, Italy; alessandro.ieraci@unimi.it

**Keywords:** BDNF, temperature, clotting duration, serum

## Abstract

Brain-derived neurothrophic factor (BDNF) is a neurotrophin expressed in different tissues and cells, including neurons, endothelial cells, leukocytes, megakaryocytes and platelets. Modifications of BDNF in plasma and/or in serum are associated with neurodegenerative and psychiatric disorders, cardiovascular diseases, metabolic syndrome and with mortality risk. Indeed, changes in blood levels of BDNF may reflect those of its tissue of origin and/or promote pathological dysfunctions. The measurement of BDNF amount in plasma or in serum has been characterized with particular attention in the impact of different anti-coagulants, clotting duration, temperature (≤21 °C) and delay in blood sample centrifugation as well as in stability of storage. However, the influences of normothermic conditions (37 °C) and of clotting duration on BDNF levels in human serum have not been investigated yet. Here, we showed that time and temperature during serum preparation could be taken into consideration to assess the association and/or impact of BDNF levels in the occurrence of pathological conditions.

## 1. Introduction

We read with great interest the article “Stability of BDNF in Human Samples Stored Up to 6 Months and Correlations of Serum and EDTA-Plasma Concentrations” published by Polyakova et al. in International Journal of Molecular Sciences on 3 June 2017 in the Special Issue, “Brain-Derived Neurotrophic Factor”. This article addressed some important issues in the evaluation of possible confounders such as pre-analytical treatment of sample and reproducibility of the analytical method in the BDNF measurement [1].

Indeed, detection of BDNF (brain-derived neurothrophic factor) levels in blood has become of great interest in the last few years in the light of its emerging role in several diseases. Modifications of circulating levels of this neurotrophin have been associated with neurodegenerative and psychiatric disorders, cardiovascular diseases, metabolic syndrome and with mortality risk [2,3,4,5]. 

BDNF may be measured both in plasma and in serum. However, BDNF levels in these two biological fluids may reflect its distinct origin and have a different physio-pathological relevance.

The measurement of BDNF levels in plasma have been well characterized with particular attention to the impact of different anti-coagulants, temperature and delay in sample centrifugation as well as in stability of sample storage [1,6,7].

Similarly, many articles focused their attention on confounders of serum BDNF levels, analyzing the influence of clotting duration, storage time and temperature conditions of samples [6,7,8,9]. Nevertheless, in these studies there is still a major open question that is the effect of clotting temperature in the levels of BDNF in serum. In particular, no information is available regarding serum BDNF kinetics at 37 °C. To maintain blood in normothermic conditions during serum preparation may be relevant in this experimental setting since BDNF is released by platelets during clotting. Indeed, in hypothermic condition, below 33 °C, not only coagulation cascade but also platelet functions can be affected, leading to a progressive delay in the thrombus formation speed [10,11,12]. 

Due to these concerns, we evaluated the influence of temperature (RT and 37 °C) on BDNF levels in human serum in relation to different clotting time-points and its release from platelets.

## 2. Results and Discussion

**Figure 1:**
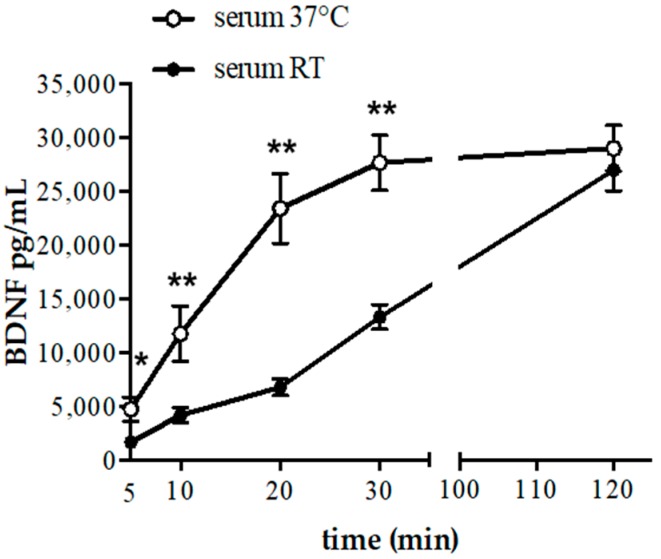
Time course of BDNF (brain-derived neurothrophic factor) concentration in serum obtained at RT (black dot) and at 37 °C (white dot). Data are expressed as mean ± SEM from 6 individuals. * *p* < 0.05 and ** *p* < 0.001.

Interestingly, the levels of BDNF in serum obtained after incubation of blood at 37 °C increased faster than in samples obtained at room temperature (RT). In particular, in samples incubated at 37 °C the plateau was reached after 30 min incubation, whereas 120 min were necessary to obtain similar BDNF levels at RT (Figure 1). It should be mentioned, however, that the use of blood collection tubes with coagulation activator might modify the BDNF kinetic in serum at room temperature bringing forward the achievement of plateau. 

Of note, the amounts of BDNF measured in re-calcified platelet rich plasma (PRP) at 37 °C for 120 min (12,512 ± 3526 pg/mL) were comparable to the values obtained at 10 min/37 °C (11,743 ± 2597 pg/mL) or 30 min/RT (13,284 ± 1132 pg/mL) (Figure 1) in the serum, suggesting that almost 60% of the total BDNF levels measured in the serum at 120 min derives from platelets. Indeed, it is well known that blood cells (e.g., leukocytes) produce and release different proteins in serum samples after 60 min of clotting [13]. Interestingly, BDNF is produced and released by circulating leukocytes [14]. 

**Figure 2:**
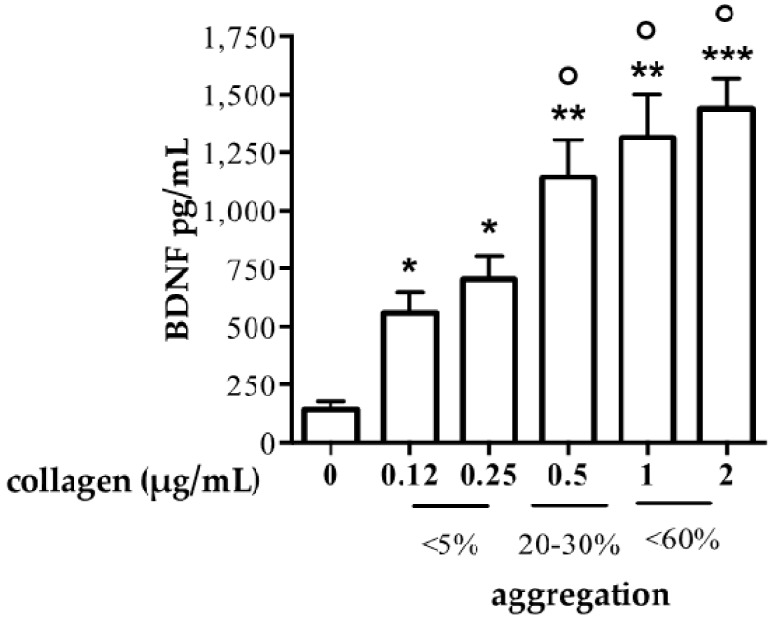
BDNF released from platelet rich plasma (PRP) activated with different concentration of collagen at 37 °C for 5 min under stirring condition (1200 rpm). Data are expressed as mean ± SEM from 6 individuals. * *p* < 0.05, ** *p* < 0.01 and *** *p* < 0.005 versus control (collagen 0 µg/mL), and ° *p* < 0.05 versus collagen 0.12–0.25 μg/mL.

In addition, low concentrations of collagen (0.12–0.25 µg/mL) were sufficient to induce BDNF release from platelets, with a mild effect on their aggregation (Figure 2). Interestingly, BDNF levels further increased reaching a plateau when aggregation was greater than 20–30% (Figure 2), suggesting that platelets degranulation, but not their aggregation, is the critical step in this process. 

In line with this evidence, in serum obtained at RT for 30 min a positive correlation between BDNF and soluble P-selectin, both released from alpha-granules, was found in patients with myocardial infarction [15]. 

Here we have showed that time and temperature during serum preparation strongly affect the levels of BDNF measured. In particular, BDNF in the serum obtained at 37 °C for 10 min as well as at RT for 30 min reflects the BDNF protein mainly released from platelets. In contrast, when serum is obtained after longer clotting time (>60 min at 37 °C or 120 min at RT), its levels in serum might reflect the total amount of BDNF produced during thrombus formation in vivo. It is important to pointing out that BDNF is usually measured in serum obtained after 1 h of clotting at RT [7,8,16]. However, according to the results presented here, the amount of BDNF measured in those experimental conditions does not correspond with platelets or total blood BDNF levels.

Thus, in order to evaluate the association and/or impact of BDNF in the occurrence of pathological conditions it is necessary to take into considerations BDNF released from both platelets and total blood. In view of these considerations, accurate timing and temperature control conditions during serum preparation are needed. 

## 3. Materials and Methods

Blood was collected by venipuncture of the antecubital vein from healthy volunteers (*n* = 6, 3 males and 3 females; mean age 33.4 ± 3.3 years who did not take antiplatelet drugs within 10 days before donation) into Vacutainer tubes containing sodium citrated and immediately centrifuged (100× *g* for 10 min at room temperature, without brake) to obtain platelet-rich plasma (PRP). Recalcified PRP (PRP with 13.8 mM of CaCl_2_) and blood collected into Vacutainer tubes with no additive and kept for 5, 10, 20, 30 and 120 min at 37 °C or at RT (23 °C) allow the blood spontaneous clotting before centrifugation (2000× *g* × 20 min at 4 °C) to obtain serum. The temperature was monitored all along experimental procedures. Platelet aggregation was performed in PRP as previously described [17]. Briefly, aggregation was induced by addition of different concentration of collagen (from 0.12 to 2 µg/mL, as indicated) and recorded for 5 min with constant stirring (1200 rpm) at 37 °C, then indomethacin (100 µM) and EDTA (5.8 mM) were added to stop the reaction and samples centrifuged at 10,000× *g* for 10 min at 4 °C. Samples and serum isolated were immediately stored at −80 °C until the analysis. Samples were stored for no longer than 1 month and BDNF levels were measured by an Emax Immunoassay system (Promega, Madison, WI, USA) as previously described [18]. 

Platelet counts were determined in citrated blood and in PRP with the Sysmex XS-1000i Hematology Analyzer (Sysmex Partec Italia s.r.l., Milan, Italy).

The study complies with the Declaration of Helsinki and was approved by the Hospital Institutional Review Board and Ethical Committee. All participants provided written informed consensum.

Statistical analyses were performed using GraphPad Prism4 software. Data were analyzed by nonparametric one- or two-way ANOVA for repeated measures followed by a Bonferroni post-hoc analysis. *p* values of less than 0.05 are considered as statistically significant. Data represent mean ± SEM.

## 4. Conclusions

In conclusion, here we have reported that time and temperature during serum preparation strongly affect the levels of BDNF measured. In particular, we suggest 30 min of blood incubation at RT or at 37 °C as the optimal duration and temperature of clotting to measure platelet or total blood BDNF concentrations, respectively.

## Abbreviations

BDNF	Brain-derived neurotrophic factor
RT	Room temperature
min	Minutes
g	Times gravity
rpm	Rounds per minute
PRP	Platelet rich plasma
EDTA	Ethylenediaminetetraacetic acid
SEM	Standard error of mean
